# THE RELATIONSHIP BETWEEN CHRONO-NUTRITION BEHAVIORS AND MUSCLE HEALTH IN OLDER ADULTS

**DOI:** 10.1093/geroni/igad104.1258

**Published:** 2023-12-21

**Authors:** Samaneh Farsijani, Ziling Mao, Peggy Cawthon, Stephen Kritchevsky, Nancy Glynn, Frederico Toledo, Anne Newman

**Affiliations:** University of Pittsburgh, Pittsburgh, Pennsylvania, United States; University of Pittsburgh, Pittsburgh, Pennsylvania, United States; California Pacific Medical Center, Research Institute, San Francisco, California, United States; Wake Forest School of Medicine, Winston Salem, North Carolina, United States; University of Pittsburgh School of Public Health, Pittsburgh, Pennsylvania, United States; University of Pittsburgh, School of Medicine, Pittsburgh, Pennsylvania, United States; University, Pittburgh, Pennsylvania, United States

## Abstract

Chrono-nutrition affects body composition via circadian clock entrainment, yet its impact on muscle health is overlooked. Here, we determined cross-sectional associations between chrono-nutrition behaviors, muscle mass (in kilogram by D3-creatine), and leg power (in Watts by Keiser) in 828 older adults (76 ± 5 years) in SOMMA. We extracted first and last intake time from 24-hr food recalls, and defined eating window as the time between first and last food/beverage intake. Chrono-nutrition’s relationships with muscle mass and power were assessed by linear regression models, adjusting for age, sex, race, energy intake, weight, height, physical activity, education, health status, study site, and marital status. Average eating window was 11 ± 2 h/d; first and last intake time were at 8:00 ± 1:00 and 19:00 ± 1:00, respectively. Eating window and last intake time were associated with muscle mass (b ± SE: 0.20 ± 0.09; 0.30 ± 0.11, respectively, P < 0.05). Longer eating window was associated with higher leg power (4.23 ± 2.14; P = 0.049). Our study found that longer eating duration is associated with higher muscle mass and power in older adults. This finding contradicts the reported benefits of intermittent fasting in promoting muscle health which has been mostly supported by non-human studies. Our findings highlight the importance of meal timing and circadian rhythms in promoting health in older adults. The impact of chrono-nutrition on health requires further investigation in different age groups and clinical settings.

